# Functional Properties of Gelatin/Polyvinyl Alcohol Films Containing Black Cumin Cake Extract and Zinc Oxide Nanoparticles Produced via Casting Technique

**DOI:** 10.3390/ijms23052734

**Published:** 2022-03-01

**Authors:** Alicja Tymczewska, Bliss Ursula Furtado, Jacek Nowaczyk, Katarzyna Hrynkiewicz, Aleksandra Szydłowska-Czerniak

**Affiliations:** 1 Department of Analytical Chemistry and Applied Spectroscopy, Faculty of Chemistry, Nicolaus Copernicus University in Toruń, Gagarina 7, 87-100 Toruń, Poland; 2 Department of Microbiology, Faculty of Biological and Veterinary Sciences, Nicolaus Copernicus University in Toruń, Lwowska 1, 87-100 Toruń, Poland; bliss.furtado@umk.pl (B.U.F.); hrynk@umk.pl (K.H.); 3 Department of Physical Chemistry and Physicochemistry of Polymers, Faculty of Chemistry, Nicolaus Copernicus University in Toruń, Gagarina 7, 87-100 Toruń, Poland; janowa@umk.pl

**Keywords:** active packaging, gelatin, polyvinyl alcohol, black cumin cake extract, zinc oxide nanoparticles, physicochemical properties, antioxidant capacity, antibacterial activity

## Abstract

This study aimed to develop and characterize gelatin/polyvinyl alcohol (G/PVA) films loaded with black cumin cake extract (BCCE) and zinc oxide nanoparticles (ZnONPs). The BCCE was also applied for the green synthesis of ZnONPs with an average size of less than 100 nm. The active films were produced by a solvent-casting technique, and their physicochemical and antibacterial properties were investigated. Supplementation of G/PVA film in ZnONPs decreased the tensile strength (TS) from 2.97 MPa to 1.69 MPa. The addition of BCCE and ZnONPs increased the elongation at the break (EAB) of the enriched film by about 3%. The G/PVA/BCCE/ZnONPs film revealed the lowest water vapor permeability (WVP = 1.14 × 10^−9^ g·mm·Pa^−1^·h^−1^·mm^−2^) and the highest opacity (3.41 mm^−1^). The QUick, Easy, New, CHEap and Reproducible (QUENCHER) methodologies using 2,2-diphenyl-1-picrylhydrazyl (DPPH), 2,2′-azino-bis(3-ethylbenzothiazoline-6- sulfonic acid) (ABTS) and cupric ion reducing antioxidant capacity (CUPRAC) were applied to measure antioxidant capacity (AC) of the prepared films. The incorporation of BCCE and ZnONPs into G/PVA films enhanced the AC by 8–144%. The films containing ZnONPs and a mixture of BCCE and ZnONPs inhibited the growth of three Gram-positive bacterial strains. These nanocomposite films with desired functional properties can be recommended to inhibit microbial spoilage and oxidative rancidity of packaged food.

## 1. Introduction

Packaging is inseparably connected to food products. Polymer-based materials are utilized as packaging to isolate the food from the surrounding environment, protect it from mechanical, physicochemical or biological degradation and prevent it from losing valuable components while extending the shelf life at the same time [1]. Petroleum-based materials dominate the food packaging marketplace due to their low cost of production and desirable material properties, which guarantee mechanical strength and thermal resistance. However, difficulties with the biodegradability and renewability of plastics require searching for alternative food packaging materials. Current consumers’ demands are concerned with natural, high-quality and safe food. Thus, there is an increasing interest in developing sustainable packaging solutions [2].

Recently, some alternatives to traditional packaging materials have been created by adding antibacterial and antioxidant substances to biopolymers (starch, cellulose, chitosan, alginate, pullulan, dairy and soy proteins and gelatin) and synthetic polymers (polyvinyl alcohol—PVA, polylactic acid—PLA, polycaprolactone—PCL) [3,4]. Motelica et al. [4] prepared alginate-based film enriched with silver nanoparticles and lemongrass essential oil as innovative packaging for cheese. Pea starch PVA films loaded with neem and oregano essential oils were proposed by Cano et al. [5] as antifungal and antibacterial novel packaging materials for food. Another study was concerned with a chitosan-acorn starch blend incorporating eugenol to improve its antioxidant and antibacterial properties [6]. However, chitosan films possess relatively poor mechanical and barrier properties. Hence, the scientific community is encouraged to develop new, active edible materials enhanced with various substances, including biopolymers and bioactive compounds.

Owing to its high availability, biocompatibility, film-forming capacity and strong mechanical and barrier properties, gelatin is one of the most frequently studied biopolymers [7]. On the other hand, gelatin-based films can be excessively rigid and brittle, generating limitations in the food-packaging field. Moreover, native gelatin materials do not possess functional properties allowing them to protect food from oxidation processes or microbial spoilage. In this context, it is crucial to improve the properties of gelatin-based materials by blending them with other polymers and incorporating antioxidant and antimicrobial substances.

Interestingly, PVA, as a nontoxic, biocompatible, biodegradable specific synthetic polymer, can be mixed with gelatin (G) to provide a stable and resistant food packaging material [3,8]. In fact, some promising G/PVA blends enhanced with various additives were developed. For instance, G/PVA copolymer film incorporated with quercetin had desirable antioxidant, thermal and morphological properties, whereas G/PVA film with mulberry anthocyanin extracts was characterized by improved elongation at break (EAB) [8,9].

Incorporation of agro-food waste and by-products into gelatin films could be an economical and environmentally friendly strategy for obtaining packaging materials with desired physicochemical properties. In fact, fruit and vegetable peels, pulps, oil cakes and shells act as natural sources of various valuable bioactive compounds (e.g., phenolic acids, carotenoids, tocopherols and vitamins) [10]. Due to this, researchers successfully incorporated various waste extracts into packaging materials. For instance, date waste extract and blood orange peel extract were used as an antioxidant additive loaded into gelatin films [11,12]. Moreover, incorporating these extracts enhanced the antioxidant and mechanical properties of materials. The addition of olive leaf extract to the gelatin matrix resulted in high antibacterial activity against a common food pathogen, *L. monocytogenes* [13].

Black cumin seed (*Nigella sativa* L.) is a popular cooking spice widely used in traditional medicine due to volatile oil and active constituents, such as thymoquinone, which exhibit high antioxidant capacity (AC). Consequently, black cumin seedcake as a by-product also constitutes a source of valuable bioactive compounds (e.g., hydroxybenzoic, syringic and p-coumaric acids). Thus, food products such as corn oil [14] and packaging materials, e.g., sago starch films [15], were enhanced with *Nigella sativa* L. seedcake extract. Moreover, this valuable by-product rich in protein was successfully used as a base for edible films [16]. Considering the present state of knowledge, extract from black cumin seedcake could become a low-priced antioxidant agent in biopolymer blends due to its nutritional value and high-value bioactive compounds.

Unfortunately, only select plant-based extracts, such as grapefruit seed extract, green tea extract or turmeric extract, can act as antimicrobial substances [17]. Therefore, the incorporation of metal or metal oxide nanoparticles characterized by strong antibacterial properties into polymeric matrices has become more popular. Researchers have lately shown positive impacts on the antimicrobial properties of gelatin blends after incorporating zinc oxide nanoparticles (ZnONPs) [18], ZnO-TiO_2_ nanoparticles [19], and Se-Ag nanoparticles [20]. Recent years have witnessed an increased interest in the application of ZnONPs in active packaging [21]. ZnONPs are easily accessible and generally recognized as safe (GRAS), approved by the Food and Drug Administration (FDA). They can be obtained using various methods, such as chemical (e.g., sol-gel or precipitation technique), physical (vapor deposition, ultrasonic irradiation) or biological (using plants, bacteria and fungus instead of chemical solvents and stabilizers). The interest in biological methods has increased dramatically in the last decade because they are deemed cost-effective and eco-friendly. In fact, ZnONPs with varied sizes from 8–400 nm and with shapes such as a polyhedron, sphere and dumbbell were successfully developed using different plant extracts from *Camellia sinensis* leaves, *Eclipta alba* leaves and *Hibiscus subdariffa* leaves [22]. Moreover, Ahmed et al. [23] demonstrated the antibacterial and antibiofilm activity of ZnONPs against the most common foodborne strains of *Escherichia coli*, *Klebsiella pneumoniae*, *Pseudomonas aeruginosa* and *Staphylococcus aureus*. A study showed antibacterial activity of *Aloe barbadensis* leaf extracts (ALE) capped with ZnO nanoparticles (ALE-ZnONPs) against *E. coli*, *P. aeruginosa* and *S. aureus* isolates. The ALE/ZnONPs inhibited the bacterial growth kinetics, exopolysaccharides and biofilm formation [24].

In the present study, the antibacterial activity of biocomposite films loaded with black cumin cake extract (BCCE) and/or ZnONPs was evaluated against the six most common foodborne bacterial pathogens in two groups: Gram-negative bacteria (*E. coli*, *K. pneumoniae* and *S. enterica*) and Gram-positive bacteria (*M. luteus*, *L. monocytogenes* and *S. aureus*).

Accordingly, the purpose of this study was to develop a functional nanocomposite based on G/PVA films doped with BCCE and ZnONPs obtained using the green approach. As shown in the preceding paragraphs, this approach is new and has not been studied so far. In this work, ZnONPs were synthesized using BCCE and characterized with the UV–Vis, X-ray diffraction (XRD) and scanning electron microscopy (SEM) methods. Importantly, BCCE and ZnONPs were both added as independent, active parts of the G/PVA films. Finally, physicochemical, mechanical, optical, morphological, antioxidant and antibacterial properties of the prepared films were investigated.

## 2. Results and Discussion

### 2.1. Characterization of Zinc Oxide Nanoparticles

**Figure 1:**
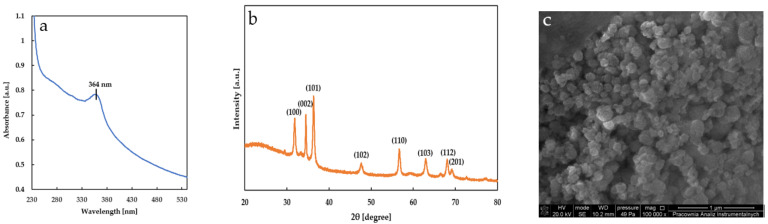
ZnO nanoparticles synthesized by black cumin cake extract (**a**) UV-Vis spectrum, (**b**) X-ray diffraction pattern (**c**) scanning electron micrograph at 100,000× magnification.

In order to confirm the formation of ZnONPs, the UV–Visible spectrum was recorded between 200–800 nm (Figure 1a).

The specific absorbance peak for ZnONPs was reported between 320 and 380 nm [25]. Thus, the absorption maximum observed at 364 nm confirmed the existence of ZnONPs.

For further confirmation, the X-ray diffraction (XRD) pattern of the obtained ZnONPs was analyzed (Figure 1b). The sharp and narrow peaks were observed at 2θ = 31.8°, 34.5°, 36.3°, 47.6°, 56.7°, 63.0°, 68.1° and 69.1°, which correspond to (100), (002), (101), (102), (110), (103), (112) and (201) planes of ZnONPs, respectively. These diffraction peaks were similar to those reported by other authors [26,27,28]. By using the Scherrer’s equation [29] (D = kλ/βcosθ, where k is constant, λ is Cu K_α_ radiation and β is peak full width at half maximum), the average particle size was calculated as 20.83 ± 6.83 nm.

The shape, structure and size of the ZnONPs synthesized using BCCE were also determined by the SEM technique. Analysis of the SEM image (Figure 1c) revealed that ZnONPs appear spherical and are partially agglomerated. This accumulation is due to the polarity and electrostatic attraction of ZnONPs. The average diameter of the particles as measured by SEM was less than 100 nm. Similarly, *Papaver somniferum* L. used for green synthesis of ZnONPs formed spherical, agglomerated nanocrystallites [30].

### 2.2. Moisture Content in Films

The results of moisture content (MC) measurements in prepared films supplemented with BCCE and ZnONPs are presented in Table 1.

According to the presented data, the MC values of the prepared films range between 18.09% and 25.63%. The MC of the control film was 18.09% and this figure increased after incorporating BCCE and ZnONPs in the G/PVA. The increase was significant for all the additives, and the highest amount of water was exhibited by the G/PVA/ZnONPs film (25.63%). A relatively high MC value in neat gelatin film can be explained by the presence of a large number of free OH groups in the gelatin matrix. Higher MC results for loaded films indicate a decrease in water-binding ability that ensures cross-linking within these films and the trapping of free water molecules via the created network by the main components of the prepared materials. ZnONPs in the film matrices exist as discontinuous and aggregated particles, making enriched films more porous with larger numbers of voids for water molecules; thus, their MC increased.

Generally, ZnONPs added to the polymer matrices decreased the MCs due to the reduction in capacity and voids in the film structure. This is actually related to the phenomenon of compaction of the structural network of the film due to the presence of nanoparticles having hydrophobic properties. On the contrary, the hydrophilicity of the ZnONPs capped with biomolecules was reported by other authors [31,32]. Shankar et al. [31] observed that all gelatin films supplemented with ZnONPs were characterized by higher MCs (12.3–14.3%) than control films (11.1%). Moreover, films made of agar, carrageenan and carboxymethyl cellulose incorporating ZnONPs possessed a higher MC (19.4, 18.2 and 19.7%, respectively) in comparison with neat films (MC = 18.7, 16.8 and 19.0%, respectively) [32].

### 2.3. Water Vapor Transmission Rate and Water Vapor Permeability of Films

The WVTR and WVP of prepared films are presented in Table 1. These parameters are important due to the substantial influence of moisture on the rate of the spoilage process of packed food. Interestingly, insignificant differences for WVTR and WVP values were observed between control film and film loaded with BCCE and with G/PVA/ZnONP material, respectively (Table 1, Newman–Keuls test). The slight increase in the WVP value of G/PVA/BCCE film was probably due to the interaction of BCCE with hydroxyl groups of the polymer chain, which weakened chain-to-chain hydrogen bonding in the polymer structure and replaced it with the new hydrogen bonds between hydroxyl groups of polymer and extract. This phenomenon increased the distance between polymer chains and introduced a twisting pore in the polymer matrix improving water vapor transmission across the film sample. Moreover, the insignificant increased WVP of the G/PVA/ZnONPs film was due to the aggregation of the nanoparticles. The aggregated ZnONPs presumably push the polymer chains apart, causing higher free volume for the water vapor to pass through more easily.

However, film with the addition of both active agents (BCCE and ZnONPs) revealed improved water-barrier properties (WVTR = 1.82 × 10^−5^ g·mm^−2^·h^−1^, WVP = 1.14 × 10^−9^ g·mm·Pa^−1^·h^−1^·mm^−2^). This suggests that BCCE in the presence of ZnONPs blocked the intrinsic hydrogen bonds in biopolymer matrix-prolonged tortuous pathways of the water vapor diffusion. Generally, WVP values of the biopolymer films decreased after the addition of ZnONPs, as was reported by Pryidarshi et al. [33], Arfat et al. [34] and Shahvalizadeh et al. [35]. On the other hand, incorporation of grapefruit seed extract could lead to a slight but not significant increase in the WVP in gelatin-based film (0.87 × 10^−9^ g·m/m^2^·Pa·s) in comparison with the control sample (0.84 × 10^−9^ g·m/m^2^·Pa·s) [36]. It is noteworthy that preparation of the film samples by the casting method may result in the formation of cracks and pores (as discussed in Section 2.6). Such ruptures appearing in different fragments of the sample could directly affect the WVP values.

### 2.4. Mechanical Properties of Films

All the samples were characterized by relatively high flexibility compared to pristine gelatin, related to the presence of PVA acting as the plasticizer in the mixtures. In the present study, film prepared from the G/PVA blend was taken as the reference material to discuss the effects of additives (BCCE and ZnONPs) on mechanical properties. As indicated by the data in Table 1, the influence of the BCCE and ZnONPs on the G/PVA blend is noticeable. Addition of the BCCE to the G/PVA blend resulted in a slight decrease in the TS (2.31 MPa) as well as the EAB (125.16%) in comparison with the control film (TS = 2.97 MPa, EAB = 137.03%). The decrease in both TS and EAB corresponds with the decrease of material toughness. Additionally, the decrease in YM resulting from including the additives shows that the films’ elasticity was raised. The BCCE contained different low molecular weight compounds that apparently decrease the intermolecular forces responsible for material cohesion. This interpretation is supported by the YM decreasing from 11.14 to 8.19 for the control and the BCCE-doped films, respectively. Consequently, the material became more susceptible to destruction while stretching. Similarly, the addition of ZnONPs led to a significant decrease in both TS (1.69 MPa) and EAB (106.46%). It is noteworthy that the addition of nanoparticles introduced a comparable YM decrease to BCCE. However, the incorporation of both the additives resulted in a drastic YM decrease from 11.14 to 6.64 MPa for G/PVA and G/PVA/BCCE/ZnONPs, respectively. This significant decrease in material toughness resulted from the substantial separation of polymeric chains by introduced additives occupying inter-chain spaces. Additionally, analysis of their morphology based on SEM images revealed considerable changes, including phase separation, which are discussed in Section 2.6. However, in the literature, the formation of weak hydrogen bonds among gelatin and ZnONPs is postulated [37]. It seems rather unlikely that this kind of interaction can occur in such a system to the extent that it would influence mechanical properties. Even if some hydrogen bonds are formed between ZnONPs and the polymer, they affect the intermolecular forces less than the physical parting of the neighboring chains by incorporated nanoparticles. Thus, the increase in intermolecular distances between chains leads to a substantial drop in material cohesion. This also is supported by the meaningful decrease in YM.

The film containing both BCCE and nanoparticles showed also decreased toughness in comparison with reference material; however, the properties are intermediate between those of the extract and ZnONPs alone. Interestingly, the EAB of this film was statistically not different from reference G/PVA but showed a significantly lower YM. To explain this characteristic, it is necessary to take into consideration morphological changes formed due to the presence of both the nanoparticles and the BCCE. The details of this are given in the discussion in Section 2.6. Summing up the most important issues, one can conclude that phase separation observed in this material causes the formation of highly porous material of different compositions of a polymeric matrix, comparing to reference. The large blobs of separated solidified phase are rare and the majority of the volume is composed of highly porous (sponge-like) polymer. Large and frequently occurring pores easily undergo deformation, which leads to a serious decrease in YM. On the other hand, this spongy phase is quite flexible and showed EAB statistically indifferent from the G/PVA reference. Additionally, it was reported in the literature that the existence of ZnONPs in polymer matrices could lead to reduced flexibility of the polymers [38,39]. However, the increase in the porosity shown in Section 2.6 explains the opposite trend observed in our materials.

Our literature survey revealed that films with similar mechanical characteristics were studied and considered as potential food packaging. Gelatin/pea protein isolate (PPI) films prepared by Hedayatnia et al. [40] possessed similar mechanical parameters (TS = 3.51–8.08 MPa, EAB = 103.1–154.6%), which were strongly affected by the presence of additional components, such as glycerol, psyllium gum and tragacanth, as well as main base polymers. Films based on gelatin/PVA reinforced with bacterial cellulose nanowhiskers, obtained by Haghighi et al. [41], were characterized by higher TS and EAB values (TS = 21.1–26.5 MPa, EAB = 28.9–40.8%). Thus, one can suspect that the studied materials have credible potential to find application in food packaging.

### 2.5. Surface Color and Optical Properties of Films

The color parameters (L, a and b) and total color difference (ΔE) of the films, based on the G/PVA blend as a control and those reinforced with BCCE and ZnONPs, are presented in Table 2.

The color parameters clearly affect consumers’ acceptance of the visual appearance of the packaging. Generally, protein-based films are colorless, depending on the concentration and type of present amino acids in the biopolymer [42]. The Newman–Keuls test indicated that the incorporation of active agents affected the color of the films’ surfaces (*p* < 0.05) by decreasing L and a values and increasing b values. In fact, lightness of the film doped with ZnONPs and BCCE was significantly reduced from 88.9 to 85.3. Shankar et al. [31] reported that the lightness of gelatin film (L = 93.30) after supplementation of ZnONPs synthesized using zinc acetate in the presence of capping agent slightly decreased (L = 92.95). On the other hand, the addition of 3% of ZnONPs into fish protein isolate/fish skin gelatin film (control film, L = 90.54) did not change its lightness significantly (L = 90.65) [34]. The a values, which determine the redness of the obtained materials, were the highest for the neat gelatin film (a = 1.0), whereas after the incorporation of BCCE and ZnONPs it decreased gradually (a ranged between 0.7 and −0.1). In contrast, the b values, indicating yellowness, increased significantly after incorporation of active agents (b changed from −4.4 to −0.6) in comparison with the control film (b = −7.5). Consequently, the highest total color difference (ΔE) was observed after blending the G/PVA mixture with BCCE and ZnONPs (ΔE = 7.9). Javidi et al. [43] also reported a decrease in the lightness of gelatin nanocomposites owing to the presence of ZnONPs and *Mentha piperita* extract. Similar observations of color changes were described by Shahvalizadeh et al. [35]. According to their results, with increased content of ZnONPs in the gelatin-tragacanth matrix, L and a values decreased, whereas b values increased (L = 91.87, a = −1.13 and b = −2.60 and L = 88.20, a = −2.73 and b = 2.13 for film without ZnONPs and film with 5% of ZnONPs, respectively).

The opacity of the obtained films ranged from 2.60 (control film) to 3.41 (G/PVA/BCCE/ZnONPs). Similar results of opacity for gelatin film (2.433) and gelatin film with 1.3 and 5% of ZnONPs (1.422 to 4.150) were reported by Sahraee et al. [44]. Moreover, there were significant differences in the opacity results for studied film samples (Table 2, Newman–Keuls test). The highest value of this parameter (3.41) was observed for the film incorporated with both active agents. An increase in the opacity values in films enriched with ZnONPs could be related to agglomeration of the nanoparticles within the gelatin/PVA matrix [45].

### 2.6. Microstructure of Films

**Figure 2:**
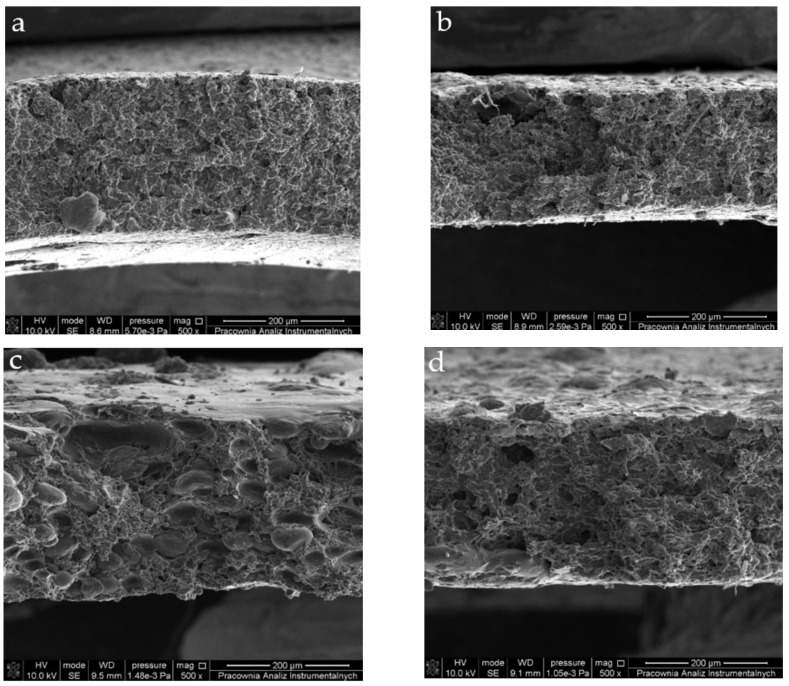
Scanning electron micrographs of cross-sections at 500× magnification of prepared gelatin/PVA films: (**a**) G/PVA (control film), (**b**) G/PVA/BCCE, (**c**) G/PVA/ZnONPs, (**d**) G/PVA/BCCE/ZnONPs.

The microstructure of the films’ cross-sections was accessed using SEM (Figure 2).

Before the main discussion of the films’ morphology, it is important to mention some facts that could be obvious for the skilled reader but should be pointed out for the sake of a clearer understanding of the materials’ structure. Firstly, it is important to note that the films were prepared applying the solution casting method; this imposed some limitations for the future morphology of the films. Secondly, it needs to be mentioned that the film-formation process was not the main concern in this study, so we did not control the casting conditions thoroughly. The film-formation process, in this case, involved aggregation, gelation and drying occurring simultaneously in a bulk steady system. The polymer chains have ample time to test new conformations before condensation in solidified form. In the meantime, solvent slowly evaporates, increasing the concentration of film precursor and facilitating gelation. As a result, the interplay of the rate of each process influence pore formation in the material. In this context, pore size, their density and distribution in the material can be controlled by solution thickness, rates of evaporation and condensation, mechanisms of gelation and many other parameters that were not analyzed during this study. Nevertheless, because all of the films were cast and dried at the same conditions, we can compare their morphological features; however, in these circumstances, it is not feasible to draw more general conclusions. Due to the formation procedure, though, all the samples revealed irregular and jagged structures with pores and cracks.

The reference G/PVA film was cast out of the solution of compatible and mixing polymers; thus, its SEM image (Figure 2a) shows a fine relatively uniform structure not revealing any sign of phase separation. The pore distribution was nearly uniform; their cross-sections were close to ellipsoid. The size of pores ranged from very small (below 0.2 µm) up to about 40 µm. Most pores (over 80%) did not exceed 1.5 µm in diameter. The film of the polymer blend with the addition of BCCE showed morphology similar to the reference with no sign of phase separation. The pore structure, however, was significantly different. Although the pores were distributed uniformly in the material volume, their size was larger. On the applied magnification 500×, the most frequent pore diameter was found to be about 3 µm. On the contrary to the G/PVA film, the pores had irregular cross-sections and a wider size distribution. As can be noticed from the SEM image (Figure 2b), the structure more closely resembled the structure of a sponge than the porous film. The SEM image of the film containing ZnONPs (Figure 2c) differed the most from the others studied. The cross-section revealed phase-separation features. Our initial explanation for this was that ZnONPs apparently had enough time during film formation to aggregate and form micrometric structures. However, Sahraee et al. [44] reported that ZnONPs are compatible and well-dispersed in a gelatin matrix. On the other hand, in the literature, one can find information on the agglomeration of ZnONPs in PVA [46], so it could be assumed that interactions of nanoparticles with PVA could be responsible for the phase separation observed in the SEM image in Figure 2c.

The aggregation leads to the formation of ellipsoidal grains with long diameters ranging from about 60 to 20 µm and short diameters between 40 and 10 µm. The presence of irregularly distributed globules of foreign phases introduces substantial disturbance in pore size and distribution. In the regions free from grains, pore size and distribution resembled the one in the reference film; however, in the vicinity of globules, pores were larger and less evenly distributed. The diameters of pores neighboring grains ranged from 5 to about 11 µm. The other film containing ZnONPs, i.e., G/PVA/BCCE/ZnONPs, showed even stronger evidence of phase separation (Figure 2d). The material was highly porous with large irregular pores. In the cross-section (Figure 2d), there was one large island of a different phase with an ellipsoidal shape over 200 µm long and about 70 µm wide. In the material, a more significant number of such inclusions were found; most of them were spotted near the film’s surface. This would suggest that some of the BCCE ingredients facilitate the phase separation in the G/PVA mixture. On the other hand, the observed phase separation is similar to some cases reported in the literature. In the case of G/PVA films, Chiellini et al. [47] reported similar cross-sections suggesting separation of one of the components from the continuous matrix formed by the second polymer. Kavoosi et al. [48] noticed that the addition of *Zataria multiflora* essential oil to the G/PVA mixture resulted in a coarse and heterogenous G/PVA film through increasing the presence of spherical particles. Consequently, samples with this essential oil revealed significantly different structures from the control film. Therefore, adding inorganic particles (ZnONPs) weakened the polymeric structure by separating constituents and forming additional defects.

### 2.7. Antioxidant Properties of Films

The antioxidant properties of the films are crucial in protecting food from oxidative processes. The ACs of the films were evaluated using the spectrophotometric QUick, Easy, New, CHEap and Reproducible (QUENCHER) methods based on direct contact of the DPPH radical (QUENCHER_DPPH_), the ABTS radical cation (QUENCHER_ABTS_) and CUPRAC reagent (QUENCHER_CUPRAC_), respectively, with the investigated film samples. The direct QUENCHER approach avoiding any pretreatment of analyzed samples is able to estimate the antioxidant potential of insoluble moiety by surface reactions occurring at the solid-liquid interface between color radicals or AC reagent and antioxidant groups bound to insoluble matter. As presented in Table 3, the QUENCHER_DPPH_, QUENCHER_ABTS_ and QUENCHER_CUPRAC_ values increased after adding the BCCE and ZnONPs compared to the neat G/PVA film.

The control film also presented antioxidant potential (QUENCHER_DPPH_ = 70.12 μmol TE/100 g, QUENCHER_ABTS_ = 207.08 μmol TE/100 g and QUENCHER_CUPRAC_ = 482.57 μmol TE/100 g). Similar observations for AC of neat gelatin films were reported in our previous paper (QUENCHER_DPPH_ = 129.42 μmol TE/100 g and QUENCHER_ABTS_ = 133.49 μmol TE/100 g) [49] as well as by Hanani [50] (DPPH = 53%). It is known that the gelatin contains antioxidant peptides or amino acids with electron-donating properties and the ability to participate in hydrogen transfer that can terminate DPPH and ABTS radical chain reactions and reduce copper (II)-neocuproine to the highly colored copper(I)-neocuproine chelate [7].

The Newman–Keuls test indicated that the AC values for the G/PVA films enriched with BCCE and ZnONPs were significantly higher than the AC values of the control film. In fact, incorporation of the BCCE resulted in the highest QUENCHER_DPPH_ and QUENCHER_CUPRAC_ values (171.09 and 756.53 μmol TE/100 g, respectively). This means that the combination of the BCCE and ZnONPs showed no synergistic effect on the antioxidant properties of the films. Although ZnONPs exhibit an ability to scavenge free radicals, their addition to the G/PVA films did not enhance the AC as much as supplementation of BCCE [51]. This could be related to an insufficient amount of the added ZnONPs to the polymeric matrix or their irregular distribution. Analogous observations were reported by Roy et al. [26]. Similar to the control sample (DPPH = 2%, ABTS = 13%), cellulose nanofiber-based film incorporated with ZnO nanorods were characterized by a negligible AC (DPPH = 1%, ABTS = 11%), whereas film enhanced with the grapefruit seed extract (GSE) possessed significantly higher DPPH (22%) and ABTS (48%) than the film supplemented with GSE and ZnONPs (DPPH = 17%, ABTS = 33%).

### 2.8. Antimicrobial Properties of Films

The antibacterial activity of films (with ZnONPs and/or BCCE) against the tested foodborne pathogens was based on the Gram character of the strains. The zone of inhibition (ZOI) clearly indicating the antibacterial effect was observed for the films loaded with ZnONPs and BCCE/ZnONPs against three Gram-positive bacterial strains. The largest inhibition zone was observed for *M. luteus* followed by *L. monocytogenes* and *S. aureus* (Table 4).

**Figure 3:**
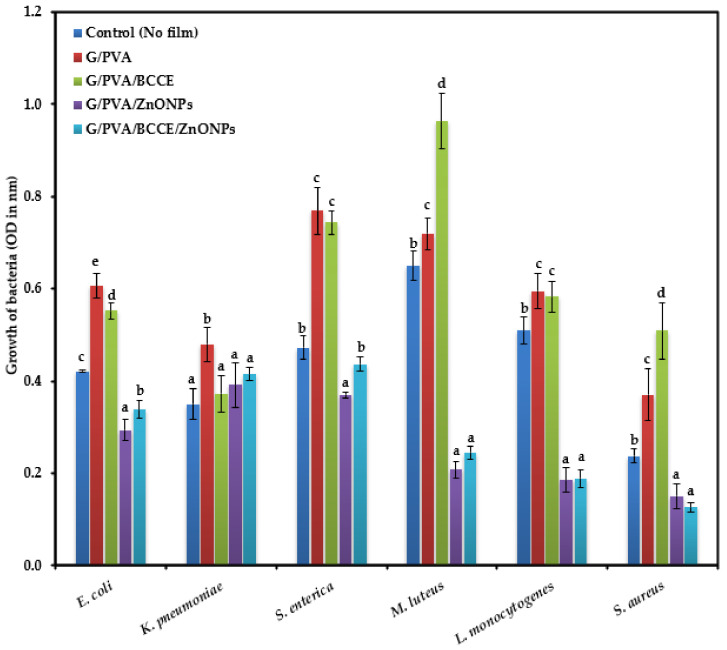
Antimicrobial activity of gelatin films enriched with BCCE and ZnONPs against Gram-negative bacteria (*E. coli*, *K. pneumoniae* and *S. enterica*) and Gram-positive bacteria (*M. luteus*, *L. monocytogenes* and *S. aureus*). Bars with different letters (a–d) represent statistical differences (one-way ANOVA and Newman–Keuls test, *p* < 0.05) between the enriched gelatin films for each of the bacteria.

The microtiter plate method showed antimicrobial activity increased in films supplemented with BCCE and ZnONPs, followed by only ZnONPs, after 24 h of incubation (Figure 3). The inhibitory effect of films enriched with ZnONPs and BCCE/ZnONPs on the Gram-negative bacteria *E. coli*, *K. pneumoniae* and *S. enterica* was less than that on the Gram-positive bacteria *M. luteus*, *L. monocytogenes* and *S. aureus*. The presence of extract in G/PVA/BCCE significantly increased the growth of the Gram-positive bacteria mainly, *M. luteus* and *S. aureus*. In addition, a higher amount of bacterial growth was observed for the three Gram-negative bacterial strains with G/PVA and G/PVA/BCCE when compared to their control (no film).

The antibacterial effect of films as observed with the disk diffusion method and microtiter plate method was more pronounced with the Gram-positive than the Gram-negative bacteria. This may indicate higher Gram-negative strain resistance/tolerance against ZnONPs than Gram-positive bacterial strains.

Similar observations were made by Premanathan et al. [52] and Azam et al. [53]; they reported that the ZnONPs’ effect was more pronounced against Gram-positive bacterial strains than Gram-negative bacterial strains. The reason for the differences in the effect of ZnONPs could be related to the cell wall characteristics of the two bacterial groups [54]. The structure of the cell wall of Gram-positive bacteria is made up of interconnected layers of peptidoglycan and lacks an outer membrane; this makes them more permeable and susceptible to toxic compounds present in the growth environment. In contrast to Gram-positive bacteria, the cell wall of Gram-negative bacteria is composed of a peptidoglycan layer and outer membrane bilayer, with lipopolysaccharides (LPS) located in the outer leaflet. Studies have shown that LPS can improve the barrier properties of an outer membrane and thus increase bacterial resistance [55]. The mechanism of ZnONPs in bacterial inhibition mainly involves Zn^2+^ particles permeating into the cell membrane, causing oxidative stress and thus damaging lipids, carbohydrates, proteins and DNA, and eventually disrupting the cellular functions [56].

Hence, we can suggest that the cell wall composition critically determines the ZnONP resistance between the two bacterial groups, making Gram-negative bacteria more resistant to ZnONPs than Gram-positive bacteria. Moreover, a significant increase in the growth of Gram-positive bacteria in the presence of extracts (G/PVA/BCCE) was seen. This is in line with our previous study where higher growth of the three Gram-positive bacterial strains was recorded in gelatin films enriched with different concentrations of water and methanolic extracts of rapeseed meal, and the growth increased with increasing concentrations of rapeseed meal extract [49].

## 3. Materials and Methods

### 3.1. Chemicals and Materials

All chemicals used in the study were of analytical or HPLC grade. Gelatin from bovine skin (20 mesh) was purchased from Chemland (Stargard Szczeciński, Poland). Polyvinyl alcohol (PVA) (molecular weight = 72000 g/mol) was purchased from Avantor Performance Materials Poland S.A. (Gliwice, Poland). Black cumin seeds in the original packaging (polyethylene film) were kindly donated by the local vegetable oil factory. Black cumin cake (BCC), the primary by-product of the black cumin oil industry, was obtained by cold pressing the seeds at room temperature with a KOMET Ca 59G screw oil expeller (MEGART, Mikołów, Poland) using a 8 mm nozzle and a rotational speed of 55 rpm.

### 3.2. Preparation of Black Cumin Cake Extract

In this study, distilled water was used for the extraction of antioxidants from BCC. A 5.0 g portion of ground BCC and 20 mL of solvent (distilled water) were transferred into round-bottomed flasks and shaken at room temperature for 30 min. Each sample was extracted in triplicate, and the residual black cumin flour was separated by centrifugation (centrifuge MPW-54, Chemland, Stargard Szczeciński, Poland, 4500 rpm, 10 min). The pooled extracts were filtered and stored in a refrigerator prior to the analysis.

### 3.3. Green Concept of Receiving Zinc Oxide Nanoparticles

Previously prepared BCC extract (V_BCCE_ = 10 mL) was transferred into the beaker and then diluted with 10 mL of distilled water. The mixture was heated and gently stirred (RH Basic 2, IKAPOL, Warszawa, Poland) until 60 °C was achieved. Then, about 3 mL of 0.1 M NaOH was added dropwise in order to reach pH = 11. After that, 1 g of zinc nitrate hexahydrate was added. The mixture was heated (60 °C) and continuously stirred for 2 h. The resulting white precipitate was filtered and washed repeatedly with distilled water followed by ethanol to remove the impurities. Finally, a white powder was obtained after overnight drying of the purified precipitate at 110 °C.

### 3.4. Characterization of Zinc Oxide Nanoparticles

#### 3.4.1. UV–Vis Spectrophotometry

The initial confirmation of ZnONPs synthesis was achieved by UV-Vis spectrophotometry using a Hitachi U-2900 spectrophotometer (Tokyo, Japan) and scanning in the range of 200–800 nm. Aqueous suspension of ZnO nano-powder for spectral was prepared according to the analysis by sonication by Kumar et al. [37] for 10 min using an ultrasonic clearer bath (5200DTD; Chemland; Stargard Szczeciński, Poland).

#### 3.4.2. X-ray Diffraction Analysis

The X-ray diffraction (XRD) of the ZnONPs was performed with a Philips X’Pert PW 3040/60 diffractometer (Philips Analytical; Almelo, The Netherlands) at room temperature in reflection mode using Cu-Kα radiation (wavelength of 1.540598 Å) at 40 kV and 40 mA. The 2θ scan data were collected at a rate of 2°/min within the scattering range of 20–80°.

#### 3.4.3. Scanning Electron Microscopy Analysis

The size and morphology of ZnONPs were examined using a SEM Quanta 3D FEG microscope (Carl Zeiss; Göttingen, Germany). The accelerating voltage used was 20 kV.

### 3.5. Preparation of Active Films

Based on preliminary experiments, the concentrations of gelatin, PVA, glycerol, BCCE and ZnONPs were determined to prepare the proposed active films. Firstly, gelatin was dissolved in distilled water (70 °C) for 20 min to obtain a concentration of 5% (*w*/*v*). PVA (5% *w*/*v*) was dissolved in water and kept under magnetic stirring at 80 °C for 2 h. A blending of the solutions (5:3 *v*/*v*) was completed by stirring for 10 min. The final filmogenic solutions were prepared using the composition given in Table 5. Gelatin, PVA, glycerol, BCCE, ZnONPs and distilled water were mixed under magnetic stirring at 60 °C for 20 min and sonicated for 2 min using an ultrasonic clearer bath. The film-forming solutions were poured into Petri dishes and left to dry at room temperature for 48 h. After drying, the films were peeled off from the casting surface.

### 3.6. Physicochemical, Optical, and Morphological Properties of Films

#### 3.6.1. Moisture Content

The MC in the obtained films was determined by the gravimetric method. A square (1 cm × 1 cm) sample was cut from each film and weighed ($W_{i}\;$—the initial weight). Then, each square was dried at 105 °C in a drying oven (SUP-3; Zalmed; Warszawa, Poland) until a constant weight was attained ($W_{f}\;$—the final dry weight). The MC in each film was analyzed in triplicate and calculated using Equation (1):

(1) $$\mathrm{MC}\;\left(\%\right)=\frac{W_{i}-W_{f}}{W_{i}}\cdot 100$$

#### 3.6.2. Water Vapor Permeability

Water vapor permeability (WVP) of the films was determined gravimetrically according to method ASTM E96-95 with slight modifications [57]. The glass cells of 29 mm in diameter containing a known mass of desiccant (silica gel, dried at 110 °C for 24 h) were prepared and sealed tightly on the top with tested films, then placed at 25 °C in a desiccator with distilled water (100% relative humidity, RH). The cells were weighed at 1 h intervals over an 8 h period. The average values of water vapor transmission rate (WVTR) and WVP were calculated based on three independent tests according to Equations (2) and (3), respectively:

(2) $$\mathrm{WVTR}\;\left(g\cdot {\mathrm{mm}}^{-2}\cdot h^{-1}\right)=\frac{w}{A\cdot t}$$

(3) $$\mathrm{WVP}\;\left(g\cdot \mathrm{mm}\cdot {\mathrm{mm}}^{-2}\cdot {\mathrm{Pa}}^{-1}\cdot h^{-1}\right)=\frac{w\cdot x}{A\cdot t\cdot \Delta P}$$

where w is the weight gained (g), t is time (h), A is area of the film exposed to water vapor permeation (mm^2^), x is the film’s average thickness (mm), and ΔP is the partial pressure difference (Pa) across the two sides of the film.

#### 3.6.3. Mechanical Properties

Mechanical properties such as the modulus of elasticity–Young’s modulus (YM), tensile strength (TS) and elongation at break (EAB) of studied films were measured according to modified ISO 527-3:2018 standard [58] using the universal testing machine Shimadzu EZ-test SX (Kyoto, Japan). The film samples were cut in rectangular strips (50 mm × 10 mm and known thickness) and conditioned at 24 °C and 55% relative humidity by placing them in a desiccator over a saturated solution of magnesium nitrate for 48 h. After this time, films were clamped between the stationary clamp connected with a load cell (100 N) with an initial separation of 30 mm, and the cross-head speed was set at 20 mm/min. The TS and EAB were measured and calculated directly from the plotted stress-strain curves.

#### 3.6.4. Surface Color and Opacity Measurement

Color parameters in the system of CIE-Lab were determined using a MICRO-COLOR II LCM 6 spectrophotometer (Dr. Bruno Lange GmbH & Co. KG, Berlin, Germany). Within the CIE-Lab system, the color is defined using lightness (L) and chromaticity parameters redness (a) and yellowness (b) of each film. The measurements were run in five replications. Additionally, the total color difference value (ΔE) was calculated using the following formula:

(4) $$\Delta E=\sqrt{[{\left(\Delta L\right)}^{2}+{\left(\Delta a\right)}^{2}+{\left(\Delta b\right)}^{2}}$$

where ΔL, Δa and Δb refer to the differences between the color value parameters of control film and G/PVA films incorporated with BCCE and ZnONPs.

The opacity of the obtained films was measured according to the method proposed by Wang et al. [59]. Each film was cut into a rectangular piece and placed in the test cell of the Hitachi U-2900 spectrophotometer (Tokyo, Japan). Then, the absorbance at 600 nm was measured five times. Film opacity was calculated by the following equation:

(5) $$\mathrm{Opacity}=\frac{Abs_{600}}{x}\;\left({\mathrm{mm}}^{-1}\right)$$

where $Abs_{600}$ is the value of absorbance at 600 nm and x is the film thickness (mm).

#### 3.6.5. Microstructure of Films

SEM cross-section imaging was performed using the Quanta 3D FEG microscope. Broken samples examined in mechanical tests were sputtered with a thin layer of gold to improve layer conductivity.

### 3.7. Antioxidant Capacity of Films

In the present study, the modified 2,2-diphenyl-1-picrylhydrazyl (DPPH), 2,2′-azino-bis(3-ethylbenzothiazoline-6-sulfonic acid) (ABTS) and cupric reducing antioxidant capacity (CUPRAC) assays previously described in detail [60] using the QUENCHER procedures were applied for direct determination of antioxidant properties of the film samples.

The AC was determined in three replications, and the results were expressed as μmol Trolox equivalents (TE) per 100 g of sample.

#### 3.7.1. QUENCHER_DPPH_ Procedure

In brief, film samples (0.1 g) were ground in an electric laboratory mill (FW100; Chemland; Stargard Szczeciński, Poland). Then, 6 mL of DPPH solution was added to the test tube containing the film sample. In the next step, samples were shaken vigorously (Classic Vortex Mixer; Velp Scientifica Srl; Usmate (MB), Italy) for 10 min to facilitate the reaction with the reagent. After shaking, test tubes were put in the dark for 15 min. After this time the absorbance of the optically clear supernatant was measured spectrophotometrically at 517 nm using a Hitachi U-2900 spectrophotometer (Tokyo, Japan).

#### 3.7.2. QUENCHER_ABTS_ Procedure

In the first step, 6 mL of ABTS radical cation solution diluted with ethanol to an absorbance of 0.70 ± 0.02 at 734 nm and weighed film sample (0.1 g) were shaken vigorously for 10 min to facilitate the reaction with the reagent. After 1 min of incubation at 40 °C, the absorbance of the optically clear supernatant was measured spectrophotometrically at 734 nm.

#### 3.7.3. QUENCHER_CUPRAC_ Procedure

In this procedure, 10 mL of mixture solution (2 mL of CuCl_2_, 2 mL of ammonium acetate buffer, 2 mL of neocuproine, 3 mL ethanol and 1 mL redistilled water) and weighed film sample (0.1 g) were shaken vigorously for 10 min to facilitate the reaction with the reagent. After 20 min of incubation at room temperature in the dark, the absorbance of the optically clear supernatant was measured spectrophotometrically at 450 nm.

### 3.8. Antibacterial Activity of Films

#### 3.8.1. Bacterial Strains and Inoculum Preparation

Six bacterial strains (*Escherichia coli*, *Klebsiella pneumoniae, Salmonella enterica, Micrococcus luteus*, *Listeria monocytogenes* and *Staphylococcus aureus*) were used to test antibacterial activity of films. The bacterial strains were cultivated in 100 mL LB broth (LB broth; Lennox; BD DifcoTM; Washington, WA, USA) and incubated at 37 °C with continuous shaking at 100 rpm for 24 h. After 24 h the bacterial cultures were centrifuged at 10,000 rpm for 10 min and the supernatant was discarded. The bacteria were washed with 10 mL of 0.9% NaCl solution and centrifuged at 10,000 rpm for 10 min. The bacterial pellets were suspended in 10 mL of sterile 0.9% NaCl solution. The optical density of each culture was adjusted to 0.07 (in sterile 0.9% NaCl solution) at 600 nm using spectrophotometer (Thermo Scientific NanoDropTM 2000/2000c Spectrophotometers; Thermo Fisher Scientific; Wilmington, DE, USA).

The bacterial suspensions were used to evaluate the antibacterial activity of the films loaded with and without BCCE and ZnONPs using the disc diffusion method and microtiter plate method.

#### 3.8.2. Antibacterial Activity by Disk Diffusion Method

The antibacterial activity of the active films were determined by the disc diffusion method [61]. 100 μL of bacterial suspension was uniformly seeded on LB agar growth medium in a Petri plate. The films were surface-sterilized under a UV lamp for 30 min on each side and aseptically cut into discs (5 mm in diameter). The film discs were placed on the surface of each bacterially seeded LB agar plate. The plates were incubated for 24 h at 37 °C. Three replicates for each bacteria and each film were maintained. The antibacterial activity of the film is shown as the diameter of inhibition zone around the disc, following the 24 h incubation [61,62].

#### 3.8.3. Antibacterial Activity by Microtiter Plate Method

The surface-sterilized films were aseptically cut into discs (5 mm in diameter). The discs were placed in each well of a sterile 96-well microtiter plate. A total of 150 μL of each bacterial suspension was pipetted into the microtiter well plates. The control wells were prepared with no film (bacterial suspension only). The final volume in each well was 150 μL. The plates were placed in an incubator set at 37 °C. The analysis was performed in four replicates. After 24 h incubation, the absorbance was measured at 600 nm using the SpectraMax iD3 microplate reader (Molecular Devices Ltd.; San Jose, CA, USA). The solutions in the wells were mixed by pipetting to ensure uniform distribution of the dissolved film in microtiter wells before measurement.

### 3.9. Statistical Analysis

The obtained results of film parameters were presented as: mean ± standard deviation (SD). One-way analysis of variance (ANOVA), followed by the Newman–Keuls test, was performed to analyze the significant differences between data (*p* < 0.05).

## 4. Conclusions

ZnONPs were successfully obtained by a green approach using extract of BCC, a residue of the oil industry. Moreover, BCCE and ZnONPs were incorporated into G/PVA films. Addition of both the active agents influenced WVP and YM values of the film the most, in comparison with the control film. The opacity was clearly affected by BCCE and ZnONP supplementation. Interestingly, AC values of the G/PVA/BCCE film, measured by QUENCHER_DPPH_ and QUENCHER_CUPRAC_ were the highest. Microbiological observations revealed that films loaded with ZnONPs possessed strong antibacterial properties against Gram-positive bacteria (*M. luteus*, *L. monocytogenes* and *S. aureus*).

This study demonstrated that incorporating BCCE and ZnONPs into G/PVA film could be a promising eco-friendly material for food packaging, replacing synthetic polymers. Furthermore, the BCC by-product of the oil industry available naturally can be used as an antioxidant additive to film materials and thus helpful in solving the problems of agricultural waste disposal.

Further research should be aimed at carrying out tests on real food systems for practical active food packaging applications.

## Figures and Tables

**Table 1:** Moisture content (MC), water vapor transmission rate (WVTR), water vapor permeability (WVP) and mechanical properties of prepared films including tensile strength (TS), elongation at break (EAB) and Young’s modulus (YM).

Film Type	MC * ± SD (%)	WVTR * ± SD (g·mm^−2^·h^−1^)	WVP * ± SD (g·mm·Pa^−1^·h^−1^·mm^−2^)	TS ** ± SD (MPa)	EAB **± SD (%)	YM ** ± SD (MPa)
G/PVA	18.09 ± 0.30 ^a^	2.45 × 10^−5^ ± 2.08 × 10^−6 b^	1.59 × 10^−9^ ± 3.93 × 10^−10 a,b^	2.97 ± 0.18 ^d^	137.03 ± 6.73 ^c^	11.14 ± 0.92 ^c^
G/PVA/BCCE	19.84 ± 0.08 ^b^	2.72 × 10^−5^ ± 2.34 × 10^−6 b^	1.93 × 10^−9^ ± 1.28 × 10^−10 b^	2.31 ± 0.08 ^c^	125.16 ± 7.13 ^b^	8.19 ± 0.97 ^b^
G/PVA/ZnONPs	25.63 ± 0.45 ^d^	2.65 × 10^−5^ ± 1.98 × 10^−6 b^	1.92 × 10^−9^ ± 1.71 × 10^−10 b^	1.69 ± 0.1 ^a^	106.46 ± 7.03 ^a^	8.51 ± 0.33 ^b^
G/PVA/BCCE/ZnONPs	24.21 ± 0.12 ^c^	1.82 × 10^−5^ ± 2.79 × 10^−6 a^	1.14 × 10^−9^ ± 1.78 × 10^−10 a^	1.84 ± 0.1 ^b^	141.45 ± 7.55 ^c^	6.64 ± 0.35 ^a^

* *n* = 3, ** *n* = 5; SD—standard deviation; different letters (^a–d^) within the same column indicate significant differences between MC, WVTR, WVP, TS, EAB and YM results of the studied films (one-way ANOVA and Newman–Keuls test, *p* < 0.05).

**Table 2:** Color and opacity properties of prepared films.

Film Type	L * ± SD	a * ± SD	b * ± SD	ΔE * ± SD	Opacity * (mm^−1^)
G/PVA	88.9 ± 0.1 ^c^	1.0 ± 0.1 ^d^	−7.5 ± 0.2 ^a^	-	2.60 ± 0.02 ^a^
G/PVA/BCCE	87.5 ± 0.2 ^b^	0.7 ± 0.1 ^c^	−4.4 ± 0.3 ^b^	3.5 ± 0.4 ^a^	2.95 ± 0.04 ^b^
G/PVA/ZnONPs	87.5 ± 0.2 ^b^	0.3 ± 0.1 ^b^	−4.1 ± 0.3 ^b^	3.8 ± 0.5 ^a^	3.29 ± 0.04 ^c^
G/PVA/BCCE/ZnONPs	85.3 ± 0.3 ^a^	−0.1 ± 0.1 ^a^	−0.6 ± 0.1 ^c^	7.9 ± 0.3 ^b^	3.41 ± 0.07 ^d^

* *n* = 5; SD—standard deviation; different letters (^a–d^) within the same column indicate significant differences between color parameters and opacity of the studied films (one-way ANOVA and Newman–Keuls test, *p* < 0.05).

**Table 3:** Antioxidant capacity of prepared films.

Film Type	AC * ± SD (μmol Trolox (TE)/100 g)		
	QUENCHER_DPPH_	QUENCHER_ABTS_	QUENCHER_CUPRAC_
G/PVA	70.12 ± 1.65 ^a^	207.08 ± 6.11 ^a^	482.57 ± 15.23 ^a^
G/PVA/BCCE	171.09 ± 4.60 ^d^	342.51 ± 5.20 ^b^	756.53 ± 1.55 ^c^
G/PVA/ZnONPs	100.50 ± 4.53 ^b^	366.49 ± 10.91 ^c^	518.97 ± 18.19 ^b^
G/PVA/BCCE/ZnONPs	116.15 ± 2.50 ^c^	391.84 ± 4.14 ^d^	733.88 ± 15.75 ^c^

* *n* = 3, SD—standard deviation; different letters (^a–d^) within the same column indicate significant differences between antioxidant properties of the studied films determined by three various analytical methods (one-way ANOVA and Newman–Keuls test, *p* < 0.05).

**Table 4:** Zone of inhibition (ZOI) produced by different films against bacterial strains. The diameter of inhibition zone is presented in mm.

Film Type	Bacterial Strain *					
	*E. coli*	*K. pneumoniae*	*S. enterica*	*M. luteus*	*L. monocytogenes*	*S. aureus*
G/PVA	No ZOI	No ZOI	No ZOI	No ZOI	No ZOI	No ZOI
G/PVA/BCCE	No ZOI	No ZOI	No ZOI	No ZOI	No ZOI	No ZOI
G/PVA/ZnONPs	No ZOI	No ZOI	No ZOI	19.3 ± 0.7 ^c^	13.2 ± 0.7 ^b^	8.3 ± 0.8 ^a^
G/PVA/BCCE/ZnONPs	No ZOI	No ZOI	No ZOI	18.0 ± 0.7 ^c^	9.9 ± 0.3 ^b^	6.7 ± 0.3 ^a^

* *n* = 6; mean ± standard deviation; different letters (^a–c^) within the same row indicate significant differences between bacteria of the studied films (one-way ANOVA and Newman–Keuls test, *p* < 0.05).

**Table 5:** Composition of film formulations.

Film Type	Gelatin (c = 5% *w*/*v*) (mL)	PVA (c = 5% *w*/*v*) (mL)	Glycerol (mL)	BCCE (mL)	ZnONPs (g)	Water (mL)
G/PVA	100	60	10	-	-	20
G/PVA/BCCE	100	60	10	10	-	10
G/PVA/ZnONPs	100	60	10	-	0.1	20
G/PVA/BCCE/ZnONPs	100	60	10	10	0.1	10

## Data Availability

The data presented in this study are available on request from the corresponding author.

## References

[1] Otoni C.G., Avena-Bustillos R.J., Azeredo H.M.C., Lorevice M.V., Moura M.R., Mattoso L.H.C., McHugh T.H. (2017). Recent Advances on Edible Films Based on Fruits and Vegetables-A Review: Fruit and Vegetable Edible Films. Compr. Rev. Food Sci. Food Saf..

[2] Mohamed S.A.A., El-Sakhawy M., El-Sakhawy M.A.-M. (2020). Polysaccharides, Protein and Lipid -Based Natural Edible Films in Food Packaging: A Review. Carbohydr. Polym..

[3] Hernández-García E., Vargas M., González-Martínez C., Chiralt A. (2021). Biodegradable Antimicrobial Films for Food Packaging: Effect of Antimicrobials on Degradation. Foods.

[4] Motelica L., Ficai D., Oprea O.-C., Ficai A., Ene V.-L., Vasile B.-S., Andronescu E., Holban A.-M. (2021). Antibacterial Biodegradable Films Based on Alginate with Silver Nanoparticles and Lemongrass Essential Oil–Innovative Packaging for Cheese. Nanomaterials.

[5] Cano A., Cháfer M., Chiralt A., González-Martínez C. (2015). Physical and Antimicrobial Properties of Starch-PVA Blend Films as Affected by the Incorporation of Natural Antimicrobial Agents. Foods.

[6] Zheng K., Xiao S., Li W., Wang W., Chen H., Yang F., Qin C. (2019). Chitosan-Acorn Starch-Eugenol Edible Film: Physico-Chemical, Barrier, Antimicrobial, Antioxidant and Structural Properties. Int. J. Biol. Macromol..

[7] Luo Q., Hossen M.A., Zeng Y., Dai J., Li S., Qin W., Liu Y. (2022). Gelatin-Based Composite Films and Their Application in Food Packaging: A Review. J. Food Eng..

[8] De Barros Vinhal G.L.R.R., Silva-Pereira M.C., Teixeira J.A., Barcia M.T., Pertuzatti P.B., Stefani R. (2021). Gelatine/PVA Copolymer Film Incorporated with Quercetin as a Prototype to Active Antioxidant Packaging. J. Food Sci. Technol..

[9] Zeng P., Chen X., Qin Y.-R., Zhang Y.-H., Wang X.-P., Wang J.-Y., Ning Z.-X., Ruan Q.-J., Zhang Y.-S. (2019). Preparation and Characterization of a Novel Colorimetric Indicator Film Based on Gelatin/Polyvinyl Alcohol Incorporating Mulberry Anthocyanin Extracts for Monitoring Fish Freshness. Food Res. Int..

[10] Dilucia F., Lacivita V., Conte A., Del Nobile M.A. (2020). Sustainable Use of Fruit and Vegetable By-Products to Enhance Food Packaging Performance. Foods.

[11] Rangaraj V.M., Rambabu K., Banat F., Mittal V. (2021). Effect of Date Fruit Waste Extract as an Antioxidant Additive on the Properties of Active Gelatin Films. Food Chem..

[12] Jridi M., Boughriba S., Abdelhedi O., Nciri H., Nasri R., Kchaou H., Kaya M., Sebai H., Zouari N., Nasri M. (2019). Investigation of Physicochemical and Antioxidant Properties of Gelatin Edible Film Mixed with Blood Orange (*Citrus sinensis*) Peel Extract. Food Packag. Shelf Life.

[13] Albertos I., Avena-Bustillos R.J., Martín-Diana A.B., Du W.-X., Rico D., McHugh T.H. (2017). Antimicrobial Olive Leaf Gelatin Films for Enhancing the Quality of Cold-Smoked Salmon. Food Packag. Shelf Life.

[14] Mariod A.A., Ibrahim R.M., Ismail M., Ismail N. (2009). Antioxidant Activity and Phenolic Content of Phenolic Rich Fractions Obtained from Black Cumin (*Nigella sativa*) Seedcake. Food Chem..

[15] Ekramian S., Abbaspour H., Roudi B., Amjad L., Nafchi A.M. (2021). Influence of *Nigella sativa* L. Extract on Physico–Mechanical and Antimicrobial Properties of Sago Starch Film. J. Polym. Environ..

[16] Sabbah M., Altamimi M., Di Pierro P., Schiraldi C., Cammarota M., Porta R. (2020). Black Edible Films from Protein-Containing Defatted Cake of *Nigella sativa* Seeds. Int. J. Mol. Sci..

[17] Olszewska M.A., Gędas A., Simões M. (2020). Antimicrobial Polyphenol-Rich Extracts: Applications and Limitations in the Food Industry. Food Res. Int..

[18] Meshram J.V., Koli V.B., Phadatare M.R., Pawar S.H. (2017). Anti-Microbial Surfaces: An Approach for Deposition of ZnO Nanoparticles on PVA-Gelatin Composite Film by Screen Printing Technique. Mater. Sci. Eng. C.

[19] Azizi-Lalabadi M., Alizadeh-Sani M., Divband B., Ehsani A., McClements D.J. (2020). Nanocomposite Films Consisting of Functional Nanoparticles (TiO_2_ and ZnO) Embedded in 4A-Zeolite and Mixed Polymer Matrices (Gelatin and Polyvinyl Alcohol). Food Res. Int..

[20] Jamróz E., Kopel P., Juszczak L., Kawecka A., Bytesnikova Z., Milosavljevic V., Makarewicz M. (2019). Development of Furcellaran-Gelatin Films with Se-AgNPs as an Active Packaging System for Extension of Mini Kiwi Shelf Life. Food Packag. Shelf Life.

[21] Kim I., Viswanathan K., Kasi G., Thanakkasaranee S., Sadeghi K., Seo J. (2020). ZnO Nanostructures in Active Antibacterial Food Packaging: Preparation Methods, Antimicrobial Mechanisms, Safety Issues, Future Prospects, and Challenges. Food Rev. Int..

[22] Bandeira M., Giovanela M., Roesch-Ely M., Devine D.M., da Silva Crespo J. (2020). Green Synthesis of Zinc Oxide Nanoparticles: A Review of the Synthesis Methodology and Mechanism of Formation. Sustain. Chem. Pharm..

[23] Ahmed B., Solanki B., Zaidi A., Khan M.S., Musarrat J. (2019). Bacterial Toxicity of Biomimetic Green Zinc Oxide Nanoantibiotic: Insights into ZnONP Uptake and Nanocolloid–Bacteria Interface. Toxicol. Res..

[24] Ali K., Dwivedi S., Azam A., Saquib Q., Al-Said M.S., Alkhedhairy A.A., Musarrat J. (2016). Aloe Vera Extract Functionalized Zinc Oxide Nanoparticles as Nanoantibiotics against Multi-Drug Resistant Clinical Bacterial Isolates. J. Colloid Sci..

[25] Agarwal H., Shanmugam V. (2020). A Review on Anti-Inflammatory Activity of Green Synthesized Zinc Oxide Nanoparticle: Mechanism-Based Approach. Bioorg. Chem..

[26] Roy S., Kim H.C., Panicker P.S., Rhim J.-W., Kim J. (2021). Cellulose Nanofiber-Based Nanocomposite Films Reinforced with Zinc Oxide Nanorods and Grapefruit Seed Extract. Nanomaterials.

[27] Vinay S.P., Chandrasekhar N. (2021). Structural and Biological Investigation of Green Synthesized Silver and Zinc Oxide Nanoparticles. J. Inorg. Organomet. Polym..

[28] Naseer M., Aslam U., Khalid B., Chen B. (2020). Green Route to Synthesize Zinc Oxide Nanoparticles Using Leaf Extracts of *Cassia fistula* and *Melia azadarach* and Their Antibacterial Potential. Sci. Rep..

[29] Bhardwaj R., Bharti A., Singh J.P., Chae K.H., Goyal N., Gautam S. (2018). Structural and Electronic Investigation of ZnO Nanostructures Synthesized under Different Environments. Heliyon.

[30] Muhammad W., Ullah N., Haroon M., Abbasi B.H. (2019). Optical, Morphological and Biological Analysis of Zinc Oxide Nanoparticles (ZnO NPs) Using *Papaver somniferum* L.. RSC Adv..

[31] Shankar S., Teng X., Li G., Rhim J.-W. (2015). Preparation, Characterization, and Antimicrobial Activity of Gelatin/ZnO Nanocomposite Films. Food Hydrocoll..

[32] Kanmani P., Rhim J.-W. (2014). Properties and Characterization of Bionanocomposite Films Prepared with Various Biopolymers and ZnO Nanoparticles. Carbohydr. Polym..

[33] Priyadarshi R., Kim S.-M., Rhim J.-W. (2021). Carboxymethyl Cellulose-Based Multifunctional Film Combined with Zinc Oxide Nanoparticles and Grape Seed Extract for the Preservation of High-Fat Meat Products. Sustain. Mater. Technol..

[34] Arfat Y.A., Benjakul S., Prodpran T., Sumpavapol P., Songtipya P. (2014). Properties and Antimicrobial Activity of Fish Protein Isolate/Fish Skin Gelatin Film Containing Basil Leaf Essential Oil and Zinc Oxide Nanoparticles. Food Hydrocoll..

[35] Shahvalizadeh R., Ahmadi R., Davandeh I., Pezeshki A., Seyed Moslemi S.A., Karimi S., Rahimi M., Hamishehkar H., Mohammadi M. (2021). Antimicrobial Bio-Nanocomposite Films Based on Gelatin, Tragacanth, and Zinc Oxide Nanoparticles—Microstructural, Mechanical, Thermo-Physical, and Barrier Properties. Food Chem..

[36] Riahi Z., Priyadarshi R., Rhim J.-W., Bagheri R. (2020). Gelatin-Based Functional Films Integrated with Grapefruit Seed Extract and TiO_2_ for Active Food Packaging Applications. Food Hydrocoll..

[37] Kumar S., Mudai A., Roy B., Basumatary I.B., Mukherjee A., Dutta J. (2020). Biodegradable Hybrid Nanocomposite of Chitosan/Gelatin and Green Synthesized Zinc Oxide Nanoparticles for Food Packaging. Foods.

[38] Ahmad A.A., Sarbon N.M. (2021). A Comparative Study: Physical, Mechanical and Antibacterial Properties of Bio-Composite Gelatin Films as Influenced by Chitosan and Zinc Oxide Nanoparticles Incorporation. Food Biosci..

[39] Amjadi S., Emaminia S., Davudian S.H., Pourmohammad S., Hamishehkar H., Roufegarinejad L. (2019). Preparation and Characterization of Gelatin-Based Nanocomposite Containing Chitosan Nanofiber and ZnO Nanoparticles. Carbohydr. Polym..

[40] Hedayatnia S., Tan C.P., Joanne Kam W.-L., Tan T.B., Mirhosseini H. (2019). Modification of Physicochemical and Mechanical Properties of a New Bio-Based Gelatin Composite Films through Composition Adjustment and Instantizing Process. LWT Food Sci. Technol..

[41] Haghighi H., Gullo M., La China S., Pfeifer F., Siesler H.W., Licciardello F., Pulvirenti A. (2021). Characterization of Bio-Nanocomposite Films Based on Gelatin/Polyvinyl Alcohol Blend Reinforced with Bacterial Cellulose Nanowhiskers for Food Packaging Applications. Food Hydrocoll..

[42] Villasante J., Martin-Lujano A., Almajano M.P. (2020). Characterization and Application of Gelatin Films with Pecan Walnut and Shell Extract (*Carya illinoiensis*). Polymers.

[43] Javidi S., Mohammadi Nafchi A., Moghadam H.H. (2021). Synergistic Effect of Nano-ZnO and *Mentha piperita* Essential Oil on the Moisture Sorption Isotherm, Antibacterial Activity, Physicochemical, Mechanical, and Barrier Properties of Gelatin Film. J. Food Meas. Charact..

[44] Sahraee S., Ghanbarzadeh B., Milani J.M., Hamishehkar H. (2017). Development of Gelatin Bionanocomposite Films Containing Chitin and ZnO Nanoparticles. Food Bioprocess Technol..

[45] Vejdan A., Ojagh S.M., Adeli A., Abdollahi M. (2016). Effect of TiO_2_ Nanoparticles on the Physico-Mechanical and Ultraviolet Light Barrier Properties of Fish Gelatin/Agar Bilayer Film. LWT Food Sci. Technol..

[46] Choudhary S., Sengwa R.J. (2018). ZnO Nanoparticles Dispersed PVA–PVP Blend Matrix Based High Performance Flexible Nanodielectrics for Multifunctional Microelectronic Devices. Curr. Appl. Phys..

[47] Chiellini E., Cinelli P., Fernandes E.G., Kenawy E.-R.S., Lazzeri A. (2001). Gelatin-Based Blends and Composites. Morphological and Thermal Mechanical Characterization. Biomacromolecules.

[48] Kavoosi G., Bordbar Z., Dadfar S.M., Dadfar S.M.M. (2017). Preparation and Characterization of a Novel Gelatin-Poly(Vinyl Alcohol) Hydrogel Film Loaded with *Zataria multiflora* Essential Oil for Antibacterial-Antioxidant Wound-Dressing Applications. J. Appl. Polym. Sci..

[49] Tymczewska A., Furtado B.U., Nowaczyk J., Hrynkiewicz K., Szydłowska-Czerniak A. (2021). Development and Characterization of Active Gelatin Films Loaded with Rapeseed Meal Extracts. Materials.

[50] Hanani Z.A.N., Yee F.C., Nor-Khaizura M.A.R. (2019). Effect of Pomegranate (*Punica granatum* L.) Peel Powder on the Antioxidant and Antimicrobial Properties of Fish Gelatin Films as Active Packaging. Food Hydrocoll..

[51] Das D., Nath B.C., Phukon P., Kalita A., Dolui S.K. (2013). Synthesis of ZnO Nanoparticles and Evaluation of Antioxidant and Cytotoxic Activity. Colloids Surf. B.

[52] Premanathan M., Karthikeyan K., Jeyasubramanian K., Manivannan G. (2011). Selective Toxicity of ZnO Nanoparticles toward Gram-Positive Bacteria and Cancer Cells by Apoptosis through Lipid Peroxidation. Nanomed. Nanotechnol. Biol. Med..

[53] Azam A., Ahmed A.S., Oves M., Khan M.S., Habib S.S., Memic A. (2012). Antimicrobial Activity of Metal Oxide Nanoparticles against Gram-Positive and Gram-Negative Bacteria: A Comparative Study. Int. J. Nanomed..

[54] Seltmann G., Holst O. (2013). The Bacterial Cell Wall.

[55] Nikaido H. (2003). Molecular Basis of Bacterial Outer Membrane Permeability Revisited. Microbiol. Mol. Biol. Rev..

[56] Lallo da Silva B., Abuçafy M.P., Berbel Manaia E., Oshiro Junior J.A., Chiari-Andréo B.G., Pietro R.C.R., Chiavacci L.A. (2019). Relationship Between Structure And Antimicrobial Activity Of Zinc Oxide Nanoparticles: An Overview. Int. J. Nanomed..

[57] (1993). Standard Test Methods for Water Vapour Transmission of Material.

[58] (2018). Plastics—Determination of Tensile Properties—Part 3: Test Conditions for Films and Sheets.

[59] Wang W., Liu Y., Jia H., Liu Y., Zhang H., He Z., Ni Y. (2017). Effects of Cellulose Nanofibers Filling and Palmitic Acid Emulsions Coating on the Physical Properties of Fish Gelatin Films. Food Biophys..

[60] Szydłowska-Czerniak A., Tułodziecka A. (2014). Antioxidant Capacity of Rapeseed Extracts Obtained by Conventional and Ultrasound-Assisted Extraction. J. Am. Oil Chem. Soc..

[61] Sun L., Sun J., Chen L., Niu P., Yang X., Guo Y. (2017). Preparation and Characterization of Chitosan Film Incorporated with Thinned Young Apple Polyphenols as an Active Packaging Material. Carbohydr. Polym..

[62] (1997). Performance Standard for Antimicrobial Disc Susceptibility Test.

